# *Triloculotrema euzeti* n. sp. (Monogenea, Monocotylidae) from the nasal tissues of the blackspotted smooth-hound *Mustelus punctulatus* (Carcharhiniformes, Triakidae) from off Tunisia

**DOI:** 10.1051/parasite/2016072

**Published:** 2016-12-22

**Authors:** Lobna Boudaya, Lassad Neifar

**Affiliations:** 1 Laboratoire de Biodiversité et Écosystèmes Aquatiques, Faculté des Sciences de Sfax, Université de Sfax BP 1171 3038 Sfax Tunisia

**Keywords:** Monocotylidae, Triakidae, *Mustelus punctulatus*, *Triloculotrema*, Mediterranean Sea

## Abstract

*Triloculotrema euzeti* n. sp. (Monogenea, Monocotylidae, Merizocotylinae) is described from the nasal tissues of the blackspotted smooth-hound *Mustelus punctulatus* collected from the coastal marine waters off Tunisia. The new parasite species is distinguished from the other two species of the genus, *T. japanicae* Kearn, 1993 and *T. chisholmae* Justine, 2009, by the morphology of the sclerotised male copulatory organ which has longitudinal ridges. The species is also characterised by its oötype with short descending and ascending limbs (long and more convoluted in the other two species). The presence of three peripheral loculi, which is the main characteristic of the genus *Triloculotrema* Kearn, 1993, is unconfirmed. This is the first description of a species of this genus in the Mediterranean Sea and the first record from a coastal shark.

## Abstract (fr)

*Triloculotrema euzeti* n. sp. (Monogenea, Monocotylidae, Merizocotylinae) est décrit des tissus nasaux de l’émissole pointillée *Mustelus punctulatus* collectée au niveau des côtes de Tunisie. La nouvelle espèce de parasite se distingue des deux autres espèces du genre, *T. japanicae* Kearn, 1993 et *T. chisholmae* Justine, 2009, par la morphologie de l’organe copulateur mâle sclérifié qui est pourvu d’arêtes longitudinales. L’espèce est également caractérisée par son ootype avec branches ascendante et descendante courtes (longues et plus contournées chez les deux autres espèces). La présence de trois loculi périphériques au niveau du hapteur, qui est la principale caractéristique du genre *Triloculotrema* Kearn, 1993, est non confirmée. Ceci est la première description d’une espèce du genre *Triloculotrema* en Méditerranée et le premier signalement chez un requin côtier.

## Introduction

Species of *Triloculotrema* Kearn, 1993 (Monocotylidae, Merizocotylinae) are reported from the nasal tissues of either triakid or squalid sharks. They can be distinguished from other members of the Monocotylidae by the presence of three peripheral loculi on the ventral surface of the haptor combined with the presence of one pair of well-developed hamuli [8]. Until now, there have been only two described species in this genus from the Pacific Ocean: *T. japanicae* Kearn, 1993 from the two triakids *Hemitriakis japanica* (Müller & Henle) off Japan and *Mustelus antarcticus* (Günther) off Tasmania, and *T. chisholmae* Justine, 2009 from the squalid *Squalus melanurus* Fourmanoir & Rivaton from off New Caledonia.

An undescribed species of *Triloculotrema* was reported from *M. punctulatus* Risso off Tunisia by Justine [16]. The sequence of its 28S rDNA was deposited in GenBank (Accession No.: AF387512) but not used in the phylogenetic analysis of Justine et al. [17]; this sequence was obtained from the material from Tunisia provided by one of the authors and corresponds to the new species we describe here. This study is part of a project on the monogeneans of the south shores of the Mediterranean Sea [1, 2, 4].

## Materials and methods

**Figure 1.**
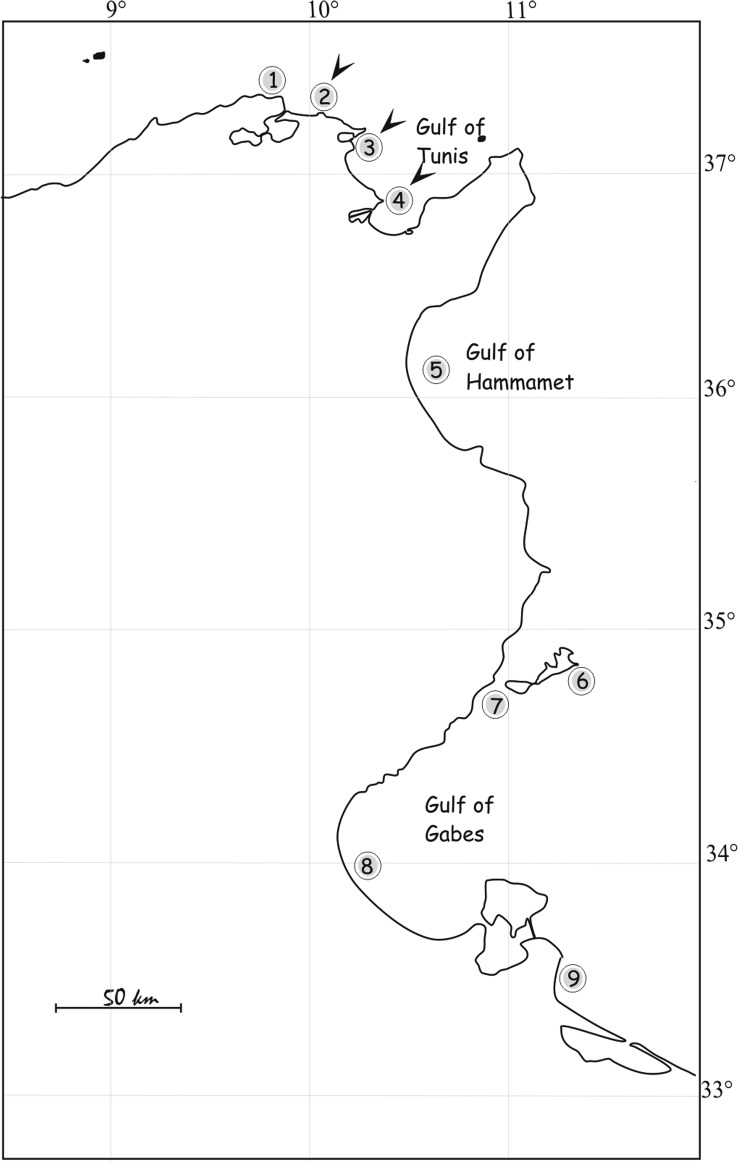
Collection sites (1–9) of *Mustelus punctulatus*. Arrows indicate where parasitised hosts were found.

The nasal fossae of 79 specimens of *M. punctulatus* (37–92 cm long) were examined between 1995 and 2015 from nine localities off Tunisia (Fig. 1). This shark is common along the Tunisian coast but seems to be more common in the south at the Gulf of Gabès [3]. Specimens were caught by local fishermen using trammel and demersal gill-nets, at depths between 10 and 100 m and were identified using keys by Compagno [9], Serena, [20] and Farrell [12]. Specimens were dissected as soon as possible after capture and the nasal tissues were removed from the cartilaginous capsule and placed in Petri dishes with filtered seawater (FSW). A small jet of FSW was used to remove excess mucus. After 15 min, the nasal tissue of each shark was inspected individually under a stereomicroscope using incident light and live worms were visible on the surface of the nasal tissue or on the bottom of the Petri dishes. Visible monogeneans were removed alive using fine dissection needles. Monogeneans were studied either directly living or fixed, slightly flattened, between a slide and coverslip. Several fixatives were used: 70% alcohol, 5% neutral formalin and Bouin-Hollande fluid. Specimens were stained with Grenacher’s carmine. After dehydration, specimens were cleared in clove oil and mounted in Canada balsam. Some parasites were fixed directly in hot 70% alcohol for scanning electron microscopy (SEM). After dehydration in an ethanol series, the material was processed in a critical point dryer (Baltec 1002) in an atmosphere of ethanol-CO_2_. The specimens were coated with gold and examined using a Philips scanning electron microscope at an accelerating voltage of 10 kV.

Drawings were made with the aid of a drawing tube using a Leitz microscope. They were scanned and redrawn on a computer using CorelDraw software. Measurements, all in micrometers, represent straight-line distances between extreme points and are expressed as the mean followed by the range and number (*n*) of structures measured in parentheses. Haptor terminology follows that of Chisholm & Whittington [7].

## *Triloculotrema euzeti* n. sp.

urn:lsid:zoobank.org:act:BC5BA193-D20F-4091-A33D-F10E68CD1097

Type-host: *Mustelus punctulatus* (Risso, 1826) (Elasmobranchii, Carcharhiniformes, Triakidae).

Site of infection: Nasal tissues.

Type-locality: Gulf of Tunis (localities 2–3–4 in Fig. 1), Tunisia (37°07′33″ N, 10°15′15″ E).

Prevalence: 16% (13 of 79 sharks infected).

Type-specimens: Holotype deposited in the Muséum National d’Histoire Naturelle, Paris as MNHN HEL588, 5 paratypes in MNHN (HEL589) and 5 paratypes in the Natural History Museum (London) (NHMUK 2016.12.20.1-5).

Etymology: Named for Pr. Louis Euzet, who had already begun the study of this species with us before his death in 2013.

### Description (Figs. 2–5)

**Figure 2.**
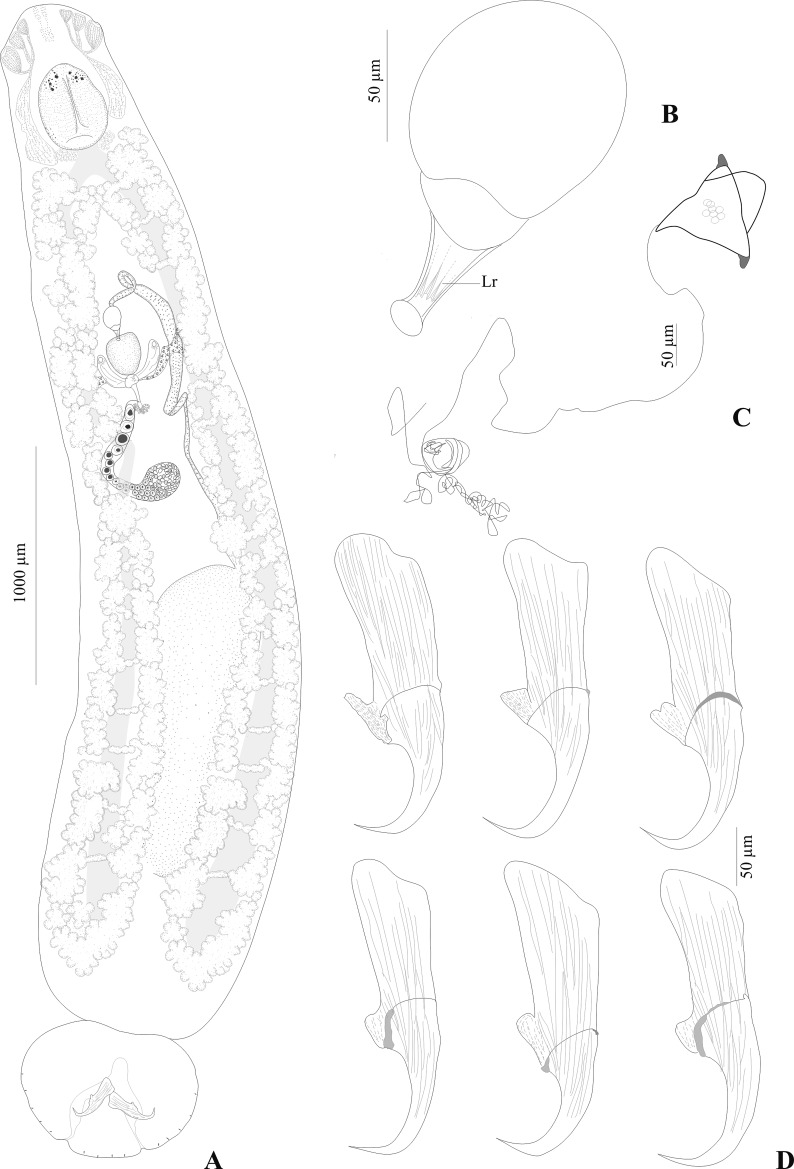
*Triloculotrema euzeti* n. sp. from *Mustelus punctulatus*. (A) Whole-mount (composite, ventral view), (B) male copulatory organ, (C) egg, (D) hamuli, variations according to different specimens. Abbreviations: Lr, longitudinal ridges.

Based on 24 whole-mounts and 3 specimens prepared for SEM. Adult body including haptor 4667 (3700–5700; *n* = 24) long, 963 (600–1450; *n* = 24) wide at level of ovary. Haptor broader than long, 605 (460–760; *n* = 13) long, 756 (560–850; *n* = 13) wide (Fig. 2A). Ventral side of haptor with indistinct loculi (see Remarks). Single pair of hamuli, 262 (225–300; *n* = 29) long. Guard of hamuli with variable morphology (Fig. 2D). Three groups of muscles attached to each hamulus. One group directed anteriorly and 2 posteriorly. Fourteen marginal hooklets, 20 (17–30; *n* = 54) long, with 2 pairs between hamuli and 5 pairs on lateral margin of haptor (Fig. 4).

**Figure 3.**
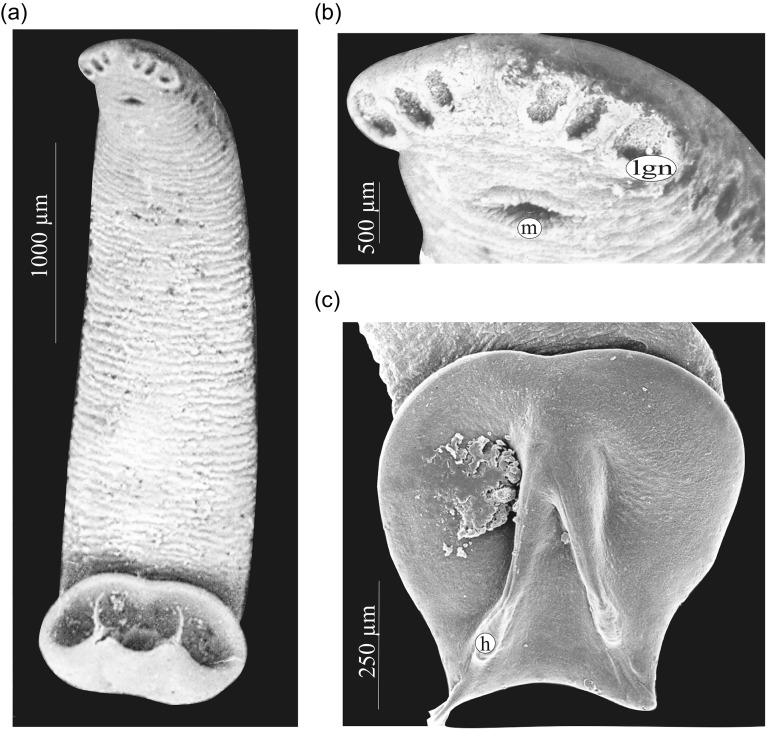
*Triloculotrema euzeti* n. sp., scanning electron micrographs. (A) Whole body (ventral view), (B) enlarged view of head region, (C) haptor. Abbreviations: h, hamulus; m, mouth; lgn, anterolateral glands containing needle-like secretion.

**Figure 4.**
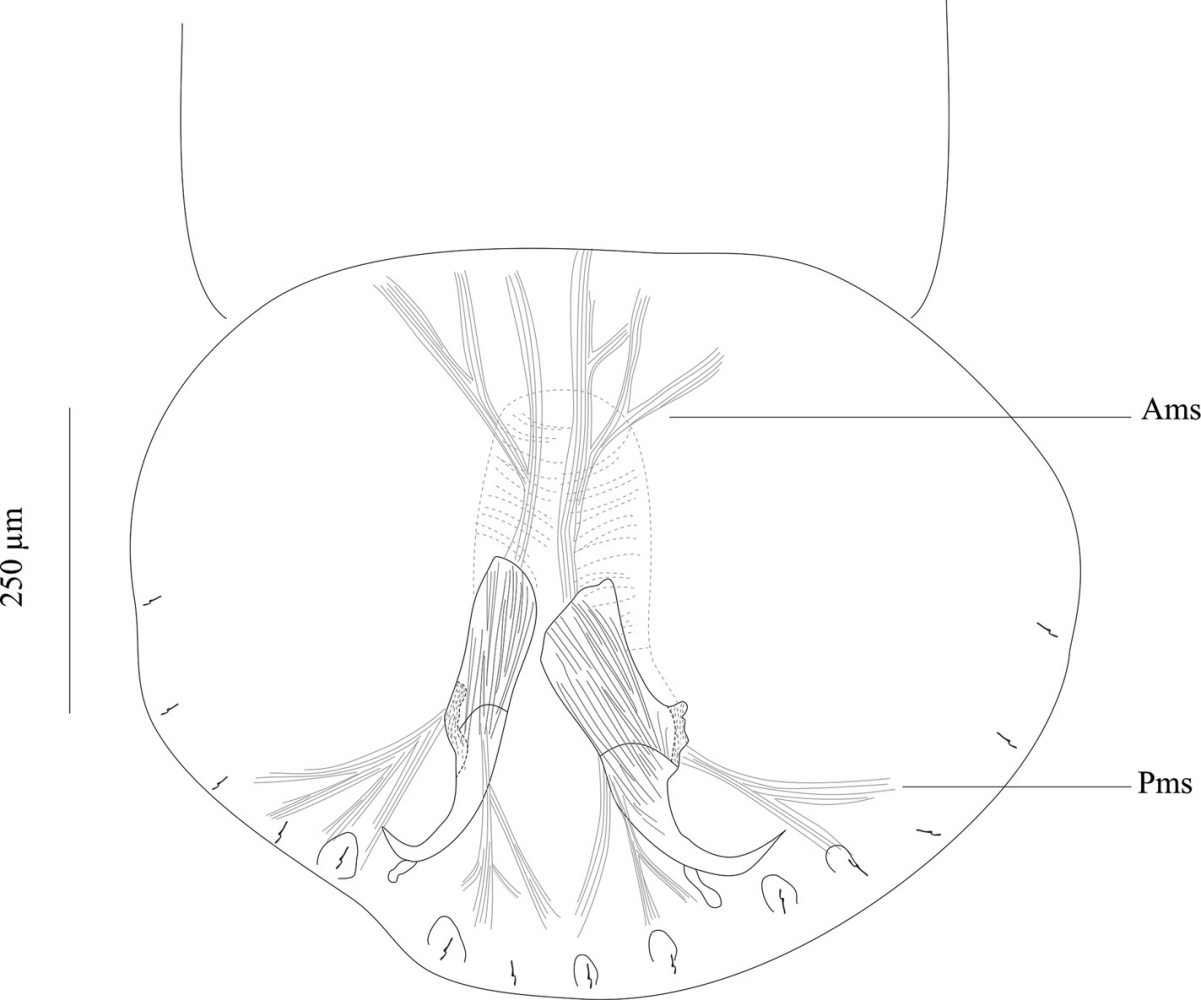
Muscles connected to the hamuli. Abbreviations: Ams, anterior muscles; Pms, posterior muscles

**Figure 5.**
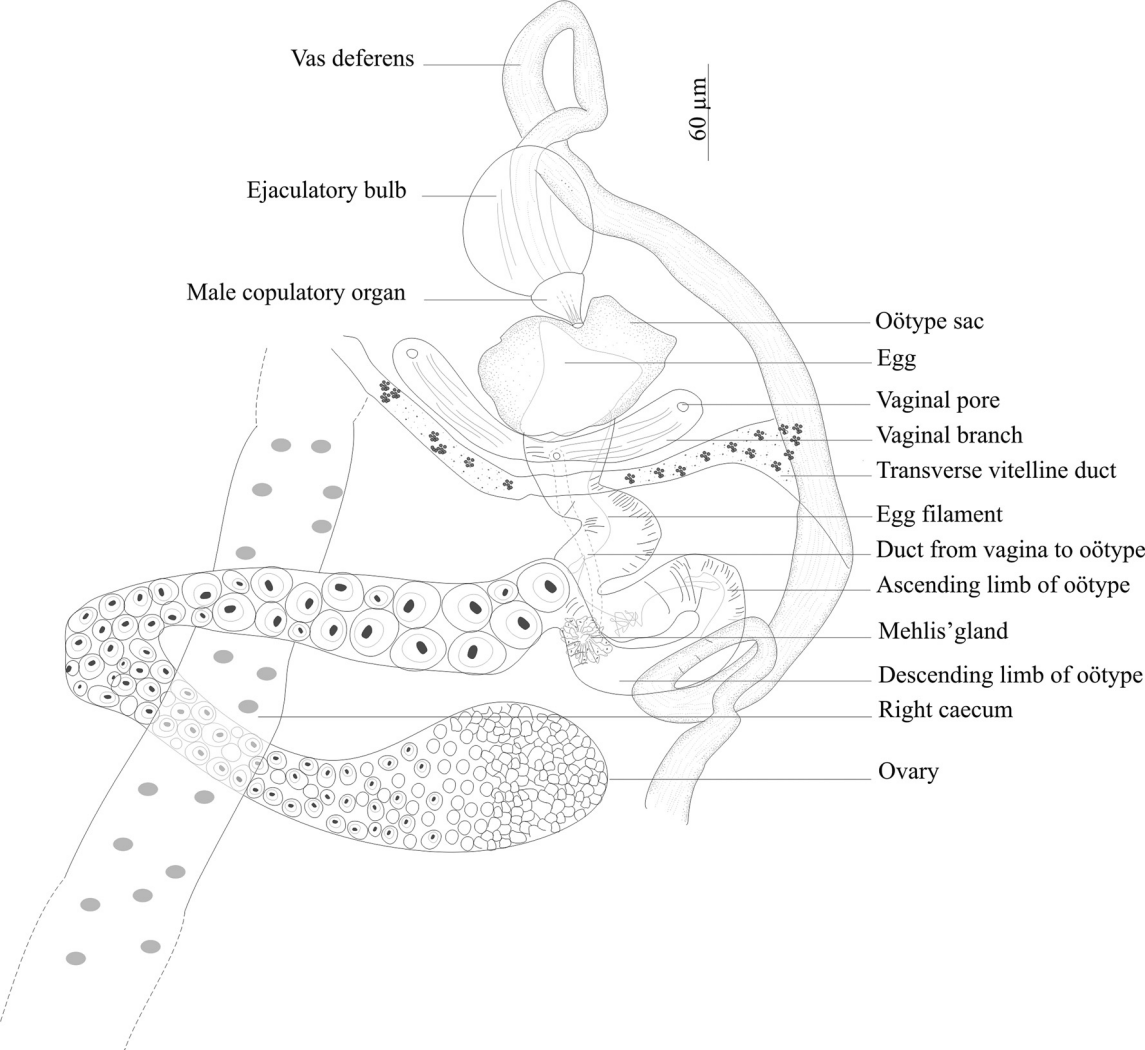
*Triloculotrema euzeti* n. sp. reproductive organs, paratype, ventral view.

Clusters of gland cells containing needle-like secretion on each side of pharynx; gland cells opening via three large anteroventral apertures on each side of the head. Apertures are progressively larger in an anterior to posterior direction (Fig. 3B). Anteromedian glands containing granular secretions present. Muscular pharynx elongate, 344 (290–460; *n* = 21) long, 264 (225–300; *n* = 21) wide. Pharyngeal glands prominent. Two intestinal caeca, inconspicuous, ending blindly posterior to testis. Scattered pigment granules present anterodorsal to pharynx.

Male copulatory organ slightly curved tube, 51 (42–55; *n* = 21) in length with longitudinal ridges (Fig. 2B). Ejaculatory bulb, 84 (71–92; *n* = 21) in diameter. Testis, single, elongate, posteriorly situated, inconspicuous, 1571 (1000–1950; *n* = 20) long, 516 (350–700; *n* = 20) wide. Vas deferens arises from antero-sinistral side of testis, runs anteriorly loops once and widens to form seminal vesicle which extends to the ejaculatory bulb (Figs. 2A, 5).

Ovary anterior to testis loops right intestinal caecum dorsoventrally and narrows to form oötype. Oötype, convoluted, forms short descending and ascending limbs and widens at anterior end (Fig. 5). Mehlis’ glands surrounding the proximal part of the oötype. Ventral openings of vaginae at the same level, just posterior to oötype sac; lateral branches from each vaginal pore fuse together to form posteriorly directed duct which connects ventrally with proximal part of oötype at the same level as Mehlis’ glands. Roughly spherical receptaculum seminis at the junction of vaginae (Fig. 2A). Eggs tetrahedral 94 (80–100; *n* = 7) long, 67 (51–95; *n* = 7) wide with posterior long polar filament (Fig. 2C). Conspicuous vitellarium, extends from the level of posterior part of pharynx to the posterior part of body proper; coextensive with intestinal caeca; occupies most of body proper. Transverse vitelline ducts join at posterior level of oötype to form medial common vitelline duct opening into oviduct (Fig. 5).

## Differential diagnosis

*Triloculotrema euzeti* n. sp. can be readily distinguished from the other two species of *Triloculotrema* by the morphology of the sclerotised male copulatory organ slightly curved and with longitudinal ridges (versus straight in *T. chisholmae* and curved in *T. japanicae*). The male copulatory organ is also shorter (42–55) in *T. euzeti* n. sp. compared with *T. japanicae* (60–64 in the original description of Kearn [18] and 73–78 in Chisholm & Whittington description [7]) but longer compared with *T. chisholmae* (40–42). The oötype with short descending and ascending limbs might represent an additional difference between *T. euzeti* and the two other species in which it is long and more convoluted. The body size is also longer (3700–5700) in *T. euzeti* compared with *T. chisholmae* (990–1260) and *T. japanicae* (2830–3110).

## Remarks

*Triloculotrema* is characterised by the presence of an oötype with long descending and ascending limbs. This character is observed in *T. japanicae* and *T. chisholmae*. However, in *T. euzeti* n. sp. the descending and ascending limbs of the oötype are very short. This might be a variable character in the genus since it is also not constant in the subfamily [7].

The two digitiform muscular processes associated with the male copulatory organ in *T. chisholmae* mentioned by Justine [16] were not seen in *T. euzeti* or in *T. japanicae* by Kearn [18] and Chisholm & Whittington [7].

The scattered pigment granules present anterodorsal to the pharynx of *T. euzeti* were also mentioned in *T. japanicae* by Kearn [18] and confirmed by Chisholm & Whittington [7] in the same species but were not seen in *T. chisholmae*. According to Justine [16], this might be a variable character in the genus. However, this could also be due to the low number of specimens (only three) observed by Justine [16].

Anteromedian glands containing granular secretions were observed in *T. euzeti*. These glands were not seen in *T. japanicae* or *T. chisholmae*. This could also be due to the low number of specimens observed for these two species (only two for *T. japanicae* and three for *T. chisholmae*). Indeed, the anterior glands were observed only in some live individuals of *T. euzeti* but were not seen in fixed material. The role of these glands is unknown, although they probably have an adhesive function [5, 11].

## Discussion

**Figure 6.**
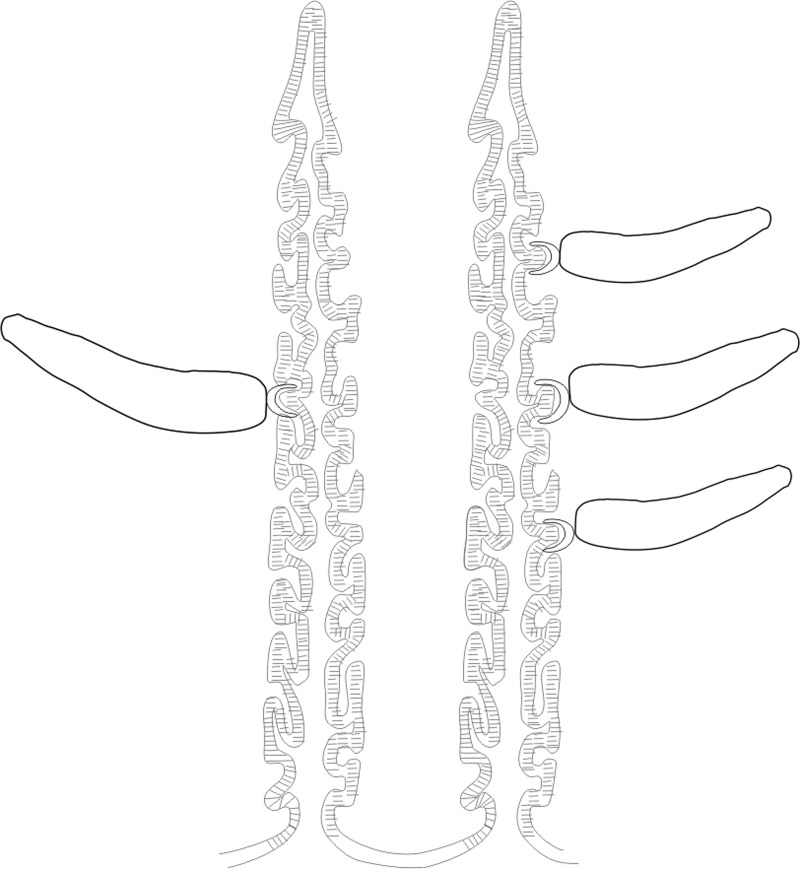
Mode of attachment of *Triloculotrema euzeti* n. sp. to the olfactory lamellae.

*Triloculotrema* was erected by Kearn [18] for *T. japanicae* from the Japanese topeshark *Hemitriakis japanicae*. Among the Monocotylidae, the most extreme reduction in the number of peripheral loculi occurs in this genus [8] characterised by the presence of three large peripheral loculi [18]. However, Justine [16] found that the loculi were not easily seen in *T. chisholmae*. In *T. euzeti* n. sp. the three loculi are also indistinct and their presence cannot be confirmed. Their limits are only suggested by muscle limits (Figs. 3C, 4). It appears in this genus that there is a trend towards the disappearance of peripheral loculi. This reduction could be related to the haptor’s mode of attachment. The haptor folds transversally to enclose the nasal lamellae and the well-developed hamuli attach the parasite by piercing the soft nasal tissue (Figs. 3A, 3C, 6). This mode of attachment seems to be effective and does not rely on the presence of loculi. When detached from the lamellae this parasite cannot attach itself to the bottom of the Petri dishes indicating the absence of haptoral loculi. Haptoral muscles are well developed indicating the important role of hamuli in the attachment. The haptoral musculature brings about a set of highly coordinated and precise movements of hamuli responsible for anchoring the parasite to the nasal lamellae of its host. Species of *Triloculotrema* live in the nasal fossae and presumably are not exposed to the same turbulence as the monocotylid species that live on the skin or gills. The nasal fossae, like the gill, have primary and secondary lamellae and water flow is unlikely to be as high as over the gills [6]. Chisholm & Whittington [7] hypothesised that, in the Merizocotylinae, the greater number of marginal loculi in the haptor is plesiomorphic and the reduction in number apomorphic. According to this hypothesis, reduction of marginal loculi number of *Triloculotrema* could be the result of the loss or fusion of loculi.

During our studies, samples of different sizes and sexes of *M. punctulatus* were collected. However, *T. euzeti* was found only in small host individuals in which the total length (TL) was less than 60 cm (males mature at 50–55 cm TL and females at 60 cm TL [10]) and were abundant in each specimen (6–11 parasites per fish). No significant differences were observed between male and female for abundance (male: 10.84; female: 11.08) and prevalence (male: 16.66; female: 16.27). This high abundance of *Triloculotrema* in small specimens could be the result of an immune response. The adaptive immune system in cartilaginous fish is slower in response [13] and could be the reason for the absence of *Triloculotrema* in adult specimens.

Samples of *M. punctulatus* from different localities along the Tunisian coast were examined in this study (Fig. 1). *T. euzeti* was collected only from the Gulf of Tunis (Fig. 1 localities 2–3–4). Intrinsic differences, such as those relating to host-parasite life history characteristics, will readily influence a species that can expand its range or invade new habitats [14]. Future studies are needed to investigate the influence of life history characteristics on the establishment of this parasite-host association.

Justine [16] assumed that species of *Triloculotrema* appear to be limited to deep-sea sharks. However, we found *T. euzeti* from a coastal shark. This demersal shark is found on the continental shelf (to 200 m depth) [20]. According to Marcogliese [19], a decrease in parasite diversity is observed with increasing depth and distance from the continental slope. In our study, the diversity of nasal monogeneans does not seem to change with depth. This preferential presence of *Triloculotrema* on triakids and squalids may be related to ecological affinities between these Chondrichthyes fishes. Most of these hosts feed on benthic or demersal prey [9] and larva infestation is likely near the bottom. Eggs have long filaments, which allow them to entangle various objects near the bottom.

*T. euzeti* is the first *Triloculotrema* recorded from the Mediterranean Sea. The other two species of the genus, *T. japanicae* and *T. chisholmae*, were described from the Pacific. It seems that *Triloculotrema* has an extended distribution and that more extensive sampling of other Triakidae and Squalidae host species from other localities around the world will yield other species of this genus.

Other monogeneans: Gračan et al. [15] recorded the presence of *Calicotyle stossichi* Braun, 1899 (Monogenea, Monocotylidae) in *M. punctulatus* from the Adriatic Sea. During our work on *M. punctulatus*, we collected *C. stossichi* from the rectal gland and *C. palombi* Euzet & Williams, 1960 from the cloaca.
